# The protective effects of chitosan and curcumin nanoparticles against the hydroxyapatite nanoparticles-induced neurotoxicity in rats

**DOI:** 10.1038/s41598-024-70794-9

**Published:** 2024-09-09

**Authors:** Gihan Mahmoud Eldeeb, Mokhtar Ibrahim Yousef, Yasser Mohamed Helmy, Hebatallah Mohammed Aboudeya, Shimaa A. Mahmoud, Maher A. Kamel

**Affiliations:** 1 Department of Environmental Studies, Institute of Graduate Studies and Research, Alexandria University, Alexandria, Egypt; 2 Pharco Company for Pharmaceutical Products, Alexandria, Egypt; 3 Department of Human Physiology, Medical Research Institute, Alexandria University, 165, Horreya Avenue, Hadara, Alexandria, Egypt; 4 Department of Biochemistry, Medical Research Institute, Alexandria University, Alexandria, Egypt; 5 Pharos University in Alexandria, Alexandria, 21311 Egypt

**Keywords:** Hydroxyapatite nanoparticles (HANPs), Curcumin nanoparticles (CUNPs), Chitosan nanoparticles (CNPs), Mitochondrial biogenesis, Oxidative stress, Neuroinflammation, Biochemistry, Neuroscience, Physiology, Nanoscience and technology

## Abstract

Hydroxyapatite nanoparticles (HANPs) have extensive applications in biomedicine and tissue engineering. However, little information is known about their toxicity. Here, we aim to investigate the possible neurotoxicity of HANPs and the possible protective role of chitosan nanoparticles (CNPs) and curcumin nanoparticles (CUNPs) against this toxicity. In our study, HANPs significantly reduced the levels of neurotransmitters, including acetylcholine (Ach), dopamine (DA), serotonin (SER), epinephrine (EPI), and norepinephrine (NOR). HANPs significantly suppressed cortical expression of the genes controlling mitochondrial biogenesis such as peroxisome proliferator activator receptor gamma coactivator 1α (PGC-1α) and mitochondrial transcription factor A (mTFA). Our findings revealed significant neuroinflammation associated with elevated apoptosis, lipid peroxidation, oxidative DNA damage and nitric oxide levels with significant decline in the antioxidant enzymes activities and glutathione (GSH) levels in HANPs-exposed rats. Meanwhile, co-supplementation of HANP-rats with CNPs and/or CUNPs significantly showed improvement in levels of neurotransmitters, mitochondrial biogenesis, oxidative stress, DNA damage, and neuroinflammation. The co-supplementation with both CNPs and CUNPs was more effective to ameliorate HANPs-induced neurotoxicity than each one alone. So, CNPs and CUNPs could be promising protective agents for prevention of HANPs-induced neurotoxicity.

## Introduction

Hydroxyapatite is a natural component of human bone and is commonly used as a bioceramic material in orthopedic and dental fields due to its excellent biocompatibility, bioactivity, similarity with the mineral components of bone and dental tissue, fusion with host material, osteoconductivity, and absence of immune response^1^. With progress in nanotechnology, hydroxyapatite nanoparticles (HANPs) were developed and used in many applications, including bone fillers, bone graft replacement, bioceramic coating, and dental fillings^2,3^. Functionalized HANPs have attracted increasing attention as new candidates for drug delivery, gene therapy, and molecular imaging applications^4^.

Although HANPs are highly promising for diverse biomedical applications, concerns about their safety are growing. NPs have been shown to penetrate and accumulate in various organs and tissues, including the brain, causing adverse biological reactions and tissue damage^5^. HANPs' potential for toxicity is currently the subject of debate and contradiction, with some research supporting their safety and others indicating toxicity in vitro and in vivo^6–8^. Previous studies confirmed the HANPs-induced nephrotoxicity in rats^9,10^. However, the neurotoxic effects of HANPs and underlying mechanisms have not yet been elucidated. As a result, there is a pressing need for the development of new protective agents to reduce HANPs-induced adverse effects and associated risks.

Apart from currently available therapeutics, traditional medicines or complementary and alternative medicines are a rich source of future drugs to treat or protect against diseases^11^. Several studies have highlighted the neuroprotective effects of curcumin and chitosan due to their antioxidant and anti-inflammatory properties^12,13^. The use of their nanoformulations has shown more efficacy in clinical applications as they are higher in solubility, bioavailability, less toxic and more deliverable into the body than free forms^14,15^. Previously, our laboratory prepared and characterized HANPs from natural bone and explored their nephrotoxicity. We used the chitosan nanoparticles (CNPs) and/or curcumin nanoparticles (CUNPs) as co-supplements to protect rat’s kidney against this toxicity and the results were promising^9,10^.

Therefore, the present study aimed to investigate the neurotoxic effects of HANPs including effects on neurotransmitters, expression of genes controlling mitochondrial biogenesis, oxidative stress markers, neuroinflammation, brain tissue architecture and proliferating cell nuclear antigen (PCNA) immunoreactivity in adult male rats. We also explored the possible protective roles of CNPs and/or CUNPs against the neurotoxicity induced by HANPs.

## Results

### Characterizations of CNPs, CUNPs and HANPs

**Fig. 1:**
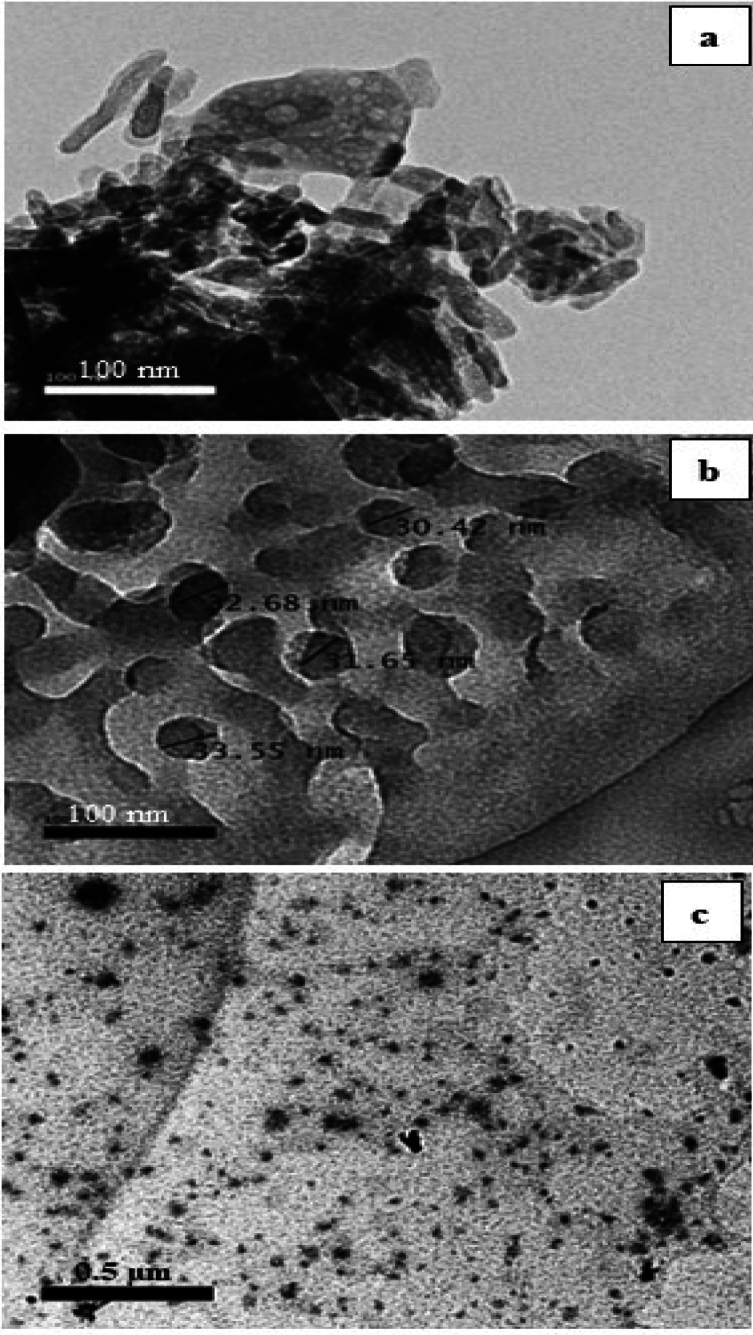
TEM images and size distribution of HANPs (**a**), CNPs (**b**) and CUNPs (**c**).

The TEM image analysis of HANPs showed agglomerated needle-like structures with an average size of 50 200 nm and a diameter of 1–2 nm (Fig. 1A), while CNPs showed smooth surfaced spherical aggregated particles with an average size of 50–100 nm and a diameter of 25–40 nm (Fig. 1B). CUNPs showed the well-defined crystalline morphology with average particle size 0.2–0.5 μm and the diameter was 0.04–0.05 μm (Fig. 1C).

### Effect of CNPs, CUNPs and HANPs and their combination on the levels of brain neurotransmitters

The levels of neurotransmitters of all groups are represented in Table 1. The normal rats treated with CUNPs alone had significantly higher levels of DA and NOR compared with control rats or rats supplemented with CNPS. CNPs combined with CUNPs significantly increased the levels of DA, SER, NOR and EPI and significantly decreased the levels of ACh compared with control rats. Treatment with HANPs showed a significant decline in the levels of all measured neurotransmitters compared with control. However, the co-administration of CNPs and/or CUNPs with HANPs significantly corrected the levels of neurotransmitters and effects were more pronounced in rats treated with the combination of CNPs, CUNPs and HANPs.

**Table 1:** The neurotransmitters levels in the cerebral cortex of control rats and rats treated with hydroxyapatite nanoparticles un-supplemented or supplemented with chitosan nanoparticles and/or curcumin nanoparticles.

	DA (pg/mg protein)	SER (ng/mg protein)	Ach (pg/mg protein)	NOR (pg/mg protein)	EPI (pg/mg protein)
Control	227 ± 4.74^d^	269 ± 5.37^b^	4.1 ± 0.22^a^	210.5 ± 1.67^c^	279 ± 5.06^b^
CNPs	35 ± 5.06^c^	270 ± 6^ab^	4 ± 0.032^ab^	198.8 ± 2.37^d^	263 ± 6.64^c^
CuNPs	247 ± 5.06^b^	271 ± 8.22^ab^	3.9 ± 0.16^b^	236.6 ± 1.90^b^	261 ± 4.42^c^
CNPS + CuNPs	257 ± 3.48^a^	277 ± 4.11^a^	3.4 ± 0.09^d^	260.7 ± 1.58^a^	327 ± 5.06^a^
HANPs	87 ± 4.74^h^	121 ± 2.21^e^	1.3 ± 0.13^f^	87.7 ± 1.45^h^	90 ± 5.69^g^
HANPS + CNPs	115 ± 5.37^g^	178 ± 4.74^d^	2.4 ± 0.13^e^	109 ± 1.04^g^	186 ± 6^f^
HANPS + CuNPs	167 ± 4.11^f^	184 ± 10.43^d^	3 ± 0.19^e^	142.6 ± 1.52^f^	196 ± 1.26^e^
HANPS + CNPS + CuNPs	201 ± 3.16^e^	237 ± 5.06^c^	3.7 ± 0.19^c^	175.7 ± 1.58^e^	238 ± 1.26^d^

Data presented as mean values ± SD and n = 10.

*ACh* Acetyl choline, *CNPs* Chitosan nanoparticles, *CUNPs* Curcumin nanoparticles, *DA* Dopamine, *EPI* Epinephrine, *HANPs* Hydroxyapatite nanoparticles, *NOR* norepinephrine, *SER* Serotonin.

Mean values within the same column not sharing common superscript letters (a, b, c, d, e, f and g) were significantly different, p < 0.05 using one-way ANOVA and Tukey post hoc test.

### Effect of CNPs, CUNPs and HANPs and their combination on the oxidative stress markers, antioxidant enzymes and oxidative DNA damage

As shown in Tables 2 and 3, supplementation of CNPs alone showed no significant effect on glutathione peroxidase (GPx), glutathione S-transferase (GST), catalase (CAT), total antioxidant capacity (TAC), nitric oxide (NO) and thiobarbituric acid reactive substances (TBARS) levels compared to control. However, treatment with CUNPs significantly increased GPx, SOD, GST, CAT, and decreased NO and produced a better effect than CNPs. The combined CNPs and CUNPs significantly induced GPx, SOD, GST, CAT, GSH, and TAC levels and reduced NO levels compared with control.

**Table 2:** The levels of glutathione, antioxidant enzymes activities and total antioxidant capacity in the cerebral cortex of control rats and rats treated with hydroxyapatite nanoparticles un-supplemented or supplemented with chitosan nanoparticles and/or curcumin nanoparticles.

	GSH (µmole/mg Protein)	GPx (mU/mg protein)	SOD (mU/mg protein)	GST (mU/mg protein)	CAT (mU/mg protein)	TAC (mmol/mg protein)
Control	26.5 ± 2.15^b^	26.5 ± 2.15^b^	36.1 ± 3.79^c^	25.1 ± 1.99^c^	23.8 ± 2.37^c^	16.7 ± 5.34^b^
CNPs	23.1 ± 2.97^c^	23.1 ± 2.97^b^	28.8 ± 5.66^d^	26.6 ± 4.65^c^	21.9 ± 1.83^cd^	17.1 ± 1.77^ab^
CuNPs	27.6 ± 2.12^b^	27.6 ± 2.12^a^	50.8 ± 2.37^b^	31.6 ± 3.98^b^	30 ± 2.97^b^	16.8 ± 4.01^b^
CNPS + CuNPs	31.7 ± 2.34^a^	31.7 ± 2.34^a^	55.3 ± 3.60^a^	37.1 ± 4.93^a^	32.2 ± 0.57^a^	19.9 ± 2.31^a^
HANPs	9.8 ± 1.74^f^	9.8 ± 1.74^d^	5.1 ± 3.32^f^	11.6 ± 2.50^e^	10 ± 2.21^g^	9.9 ± 2.43^d^
HANPS + CNPs	4.4 ± 2.72^e^	14.4 ± 2.72^d^	20.1 ± 1.93^e^	13.5 ± 3.25^e^	14 ± 2.75^f^	11.2 ± 1.39^cd^
HANPS + CuNPs	19.1 ± 2.78^d^	19.1 ± 2.78^c^	22.3 ± 2.50^e^	20.2 ± 6.23^d^	18.8 ± 2.72^e^	12.8 ± 1.58^cd^
HANPS + CNPS + CuNPs	19.3 ± 1.26^d^	19.3 ± 2.75^cd^	26.2 ± 2.94^d^	18.6 ± 3.73^d^	20 ± 2.31^de^	14.3 ± 2.02^bc^

Data presented as mean values ± SD and n = 10.

*CAT* Catalase, *CNPs* Chitosan nanoparticles, *CUNPs* Curcumin nanoparticles, *HANPs* Hydroxyapatite nanoparticles, *GPx* Glutathione peroxidase, *GSH* Glutathione, *GST* Glutathione-S-transferase, *SOD* Superoxide dismutase, *TAC* Total Antioxidant Capacity.

Mean values within the same column not sharing common superscript letters (a, b, c, d, e, f and g) were significantly different, p < 0.05 using one-way ANOVA and Tukey post hoc test.

**Table 3:** The levels of thiobarbituric acid-reactive substances, nitric oxide end products, 8-OH-2-deoxygunosine in the cerebral cortex of control rats and rats treated with hydroxyapatite nanoparticles un-supplemented or supplemented with chitosan nanoparticles and/or curcumin nanoparticles.

	TBARS (nmol/mg protein)	NOx (μmol/mg protein)	8-OHdG (pg/μg DNA)
Control	5.8 ± 2.46^e^	87.4 ± 2.12^e^	12.4 ± 2.50^c^
CNPs	5.8 ± 0.63^e^	85.2 ± 3.51^e^	11.4 ± 3.10^c^
CuNPs	5.3 ± 0.73^e^	74.3 ± 5.28^f^	12.5 ± 2.21^c^
CNPS + CuNPs	8.7 ± 1.58^cd^	72.6 ± 4.08^f^	11.4 ± 3.38^c^
HANPs	15.1 ± 2.12^a^	167.6 ± 3^a^	18.1 ± 11.31^c^
HANPS + CNPs	12.1 ± 2.28^b^	138 ± 3.95^b^	15.3 ± 2.91^a^
HANPS + CuNPs	9.8 ± 1.45^c^	122.5 ± 2.34^c^	16.2 ± 2.84^b^
HANPS + CNPS + CuNPs	7.6 ± 0.76^d^	105 ± 4.96^d^	12.8 ± 3.03^ab^

Data presented as mean values ± SD and n = 10.

*8-OHdG* 8-hydroxy-2-deoxyguanosine, *CNPs* Chitosan nanoparticles, *CUNPs* Curcumin nanoparticles, *HANPs* Hydroxyapatite nanoparticles, *NOx* nitric oxide end products, *TBARS* Thiobarbituric acid reactive substances.

Mean values within the same column not sharing common superscript letters (a, b, c, d, e, f and g) were significantly different, p < 0.05 using one-way ANOVA and Tukey post hoc test.

On the other hand, the treatment with HANPs alone caused a significant decline in GPX, SOD, GST, CAT, GSH and TAC levels and a significant elevation in NO and TBARS levels compared to control. However, the combination of CNPs and CUNPs with HANPs caused a significant elevation in GPX, SOD, GST, CAT, GSH and TAC levels and a significant reduction of NO and TBARS levels compared with HANPs alone treated group.

As shown in Table 3, there was no significant difference in 8-OH-2-deoxygunaine (8-OHdG) level in CUNPs or CNPs alone and CNPS + CUNPs treated groups compared to control. On the other hand, HANPs supplementation alone increased the levels of 8-OHdG in comparison to control, while CNPs or CUNPs supplementation alone or combined with HANPs significantly reduced the brain content of 8-OHdG, though the levels remain significantly higher than control values. The treatment with the combination of CNPs, CUNPs and HANPs resulted in a complete normalization of 8-OHdG levels.

### Effect of CNPs, CUNPs and HANPs and their combination on citrate synthase (CS) activity

**Fig. 2:**
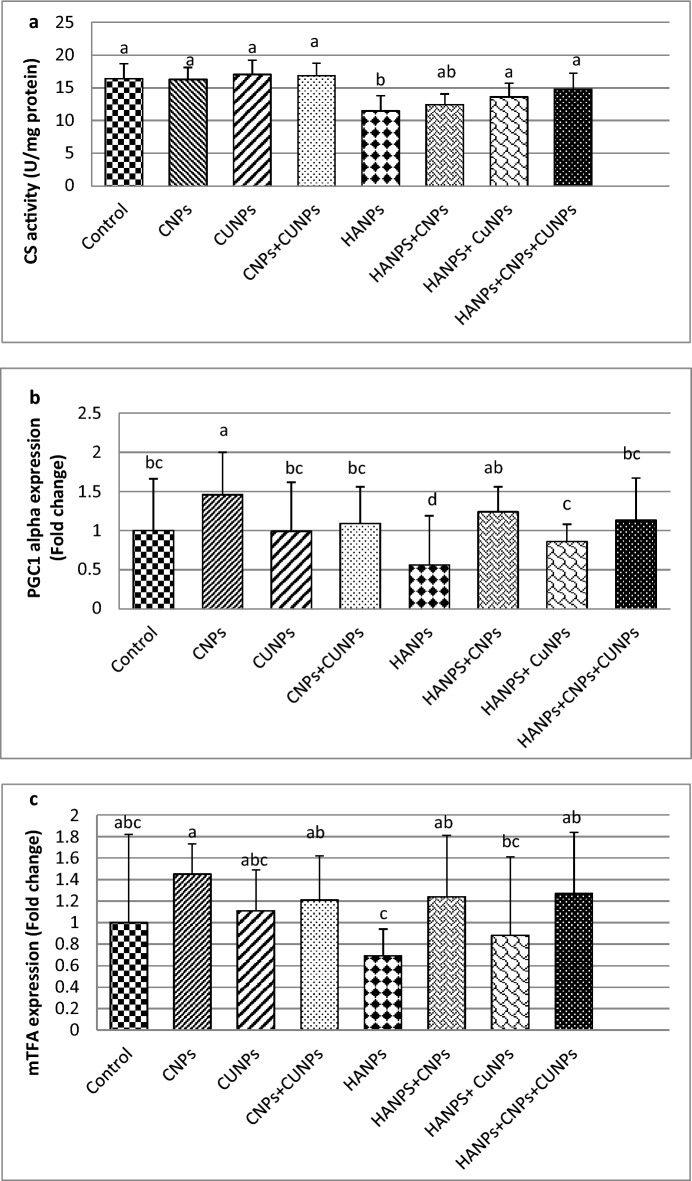
The citrate synthase (CS) activity (**a**) and gene expressions of peroxisome proliferator activator receptor gamma-coactivator 1α (PGC-1α) (**b**) and mitochondrial transcription factor A (mTFA) (**c**) in the brain tissues of male rats treated with nanoparticles of chitosan (CNPs), curcumin (CUNPs) and hydroxyapatite (HANPs) and their combination.

As shown in Fig. 2a, HANPs supplementation reduced the levels of CS activity compared to control and this was reversed by CUNPs supplementation alone or combined with CNPs and HANPs.

### Effect of CNPs, CUNPs and HANPs and their combination on the gene expressions of peroxisome proliferator activator receptor gamma-coactivator 1α (PGC-1α) and mitochondrial transcription factor A (mTFA)

The results of brain mRNA expression of PGC-1α and mTFA of the studied groups are shown in Fig. 2. The rats supplemented with CNPs alone had significantly higher expression level of PGC-1α compared to control, while the rats treated with CUNPs alone or in combination with CNPs revealed no significant effect on PGC-1α expression in comparison to control rats. Furthermore, the supplementation of rats with HANPs alone resulted in significant suppression of brain mRNA expression of PGC-1α by about 44%. The co-administration of CNPs and/or CUNPs with HANPs significantly induced PGC-1α expression.

Regarding mTFA mRNA expression, the rats supplemented with CNPs alone or combined with CUNPs significantly increased mTFA expression compared to control, while treatment with CUNPs alone showed no significant effect. The HANPs supplementation significantly suppressed the mTFA mRNA expression by about 31% compared with control. The presence of CNPs alone in combination with HANPs significantly increased mTFA expression to be 1.24-fold over control, while the presence of CUNPs alone with HANPs significantly increased its expression to 0.88-fold over control. The co-administration of CNPs and CUNPs with HANPs significantly increased the expression of mTFA to 1.27-fold over control.

### Effect of CNPs, CUNPs and HANPs and their combination on the pro-inflammatory cytokines and tumor suppressor gene p53

**Fig. 3:**
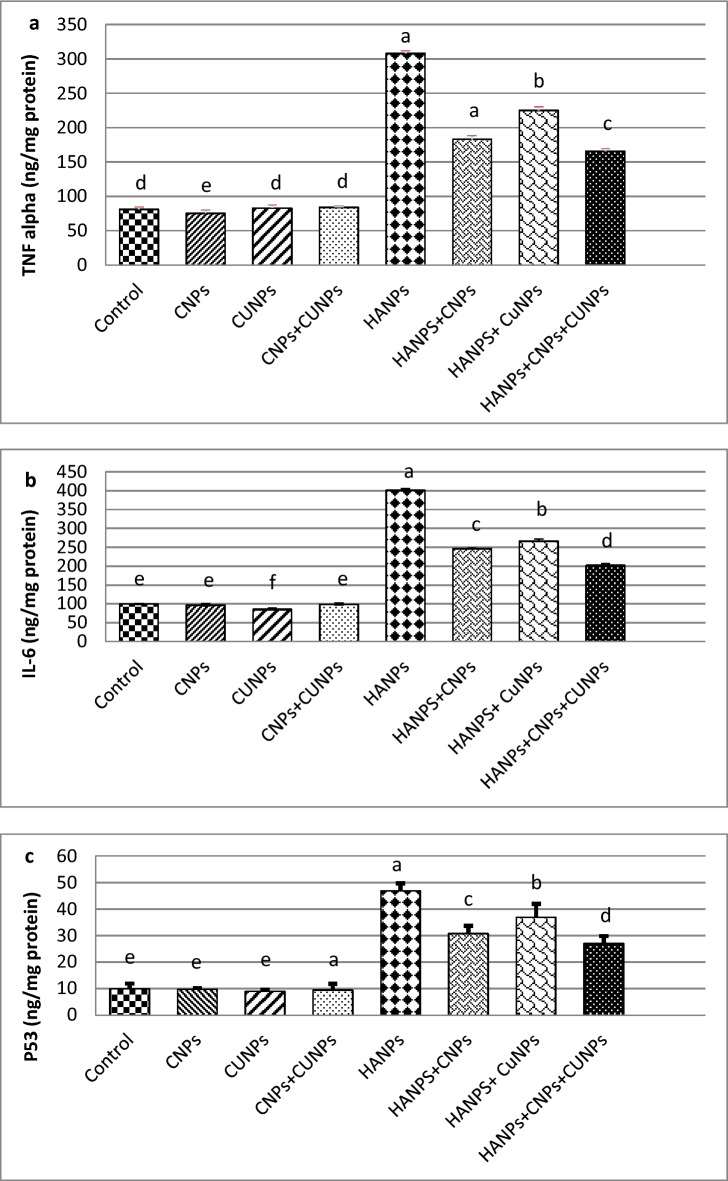
The levels of brain tumor necrosis factor-α (TNF-α) (**a**), interliukin-6 (IL-6) (**b**) and tumor suppressor (P53) (**c**) of male rats treated with nanoparticles of chitosan (CNPs), curcumin (CUNPs) and hydroxyapatite (HANPs ) and their combination.

The TNF-α, IL-6 and p53 levels in the brain tissues of all groups, are shown in Fig. 3. Findings revealed a significant reduction in TNF-α levels in CNPs alone treated group, while a significant decrease in the levels of IL-6 was detected in CUNPs alone treated group in comparison to control. Both nanoantioxidants have no significant effect on p53 levels. Also, the combination of CNPs and CUNPs had no significant effect on the levels of these parameters. The HANPs alone treated group showed a significant elevation in TNF-α, IL-6 and p53 levels compared to control. The combination of CNPs and CUNPs with HANPs led to a significant decline in the levels of these parameters as compared with HANPs treated group with a better effect observed in the CNPs + HANPs group. The combination of CNPs, CUNPs and HANPs induced an improvement in TNF-α, IL-6 and p53 levels, though the levels remain significantly higher than the values of control group.

### Effect of CNPs, CUNPs and HANPs and their combination on the brain tissues histopathology

**Fig. 4:**
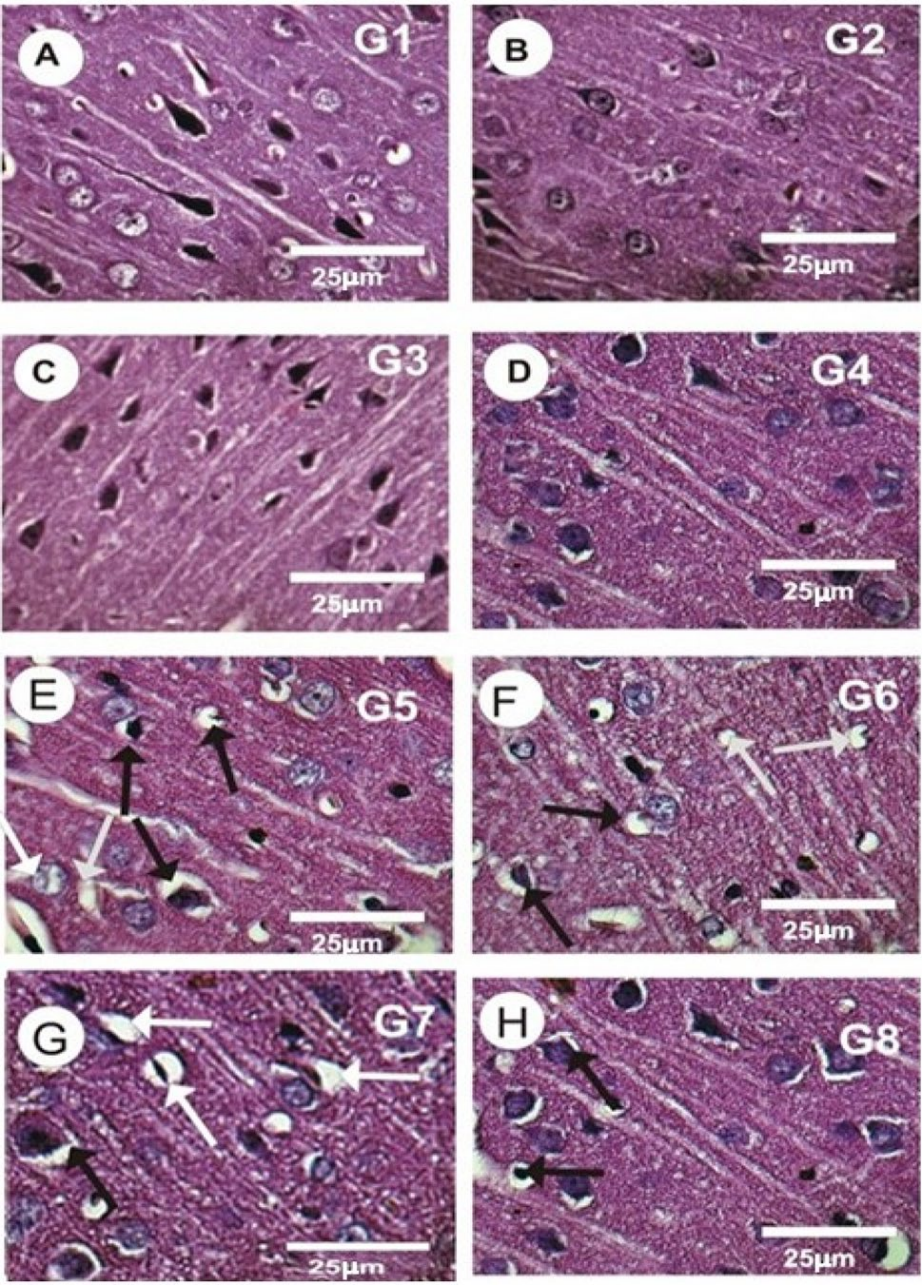
Photomicrographs of rat brain cortex sections in the different experimental groups stained with Haematoxylin & Eosin. (**A**–**D**) Rat brain cortex sections in control (G1), treated rats with CNPs (G2), with CUNPs (G3) and with CNPs and CUNPs together (G4) revealed normal neuronal structure of the neurons and nerve fibers. (**E**) Brain cortex sections in treated rats with hydroxyapatite nanoparticles revealed the presence of a large number of damages neurons (Black arrow), marked atrophied, moderate degenerating neurons, many of apoptotic bodies, and moderate neuronal atrophy (White arrow). (**F**) Brain cortex sections in treated HANPs and CNPs (G6) showed moderate number of damages neurons (Black arrow), mild neuronal atrophy (White arrow) and moderate neurofibrillary degeneration. (**G**) Brain sections in treated rat with HANPs and CUNPs (G7) revealed moderate degenerating neurons, apoptotic bodies and mild neuronal atrophy (Black arrow). (**H**) Brain sections in treated HANPs, CNPs and CUNPs all together (G8) showed a more or less normal distribution of the neurons and nerve fibers with only mild atrophy (Black arrow).

The histological results of the brain sections of control rats (G1), rats treated with CNPs (G2), or with CUNPs (G3), or with both CNPs and CUNPs (G4) revealed normal structure of neurons and nerve fibers (Fig. 4A–D). In contrast, the brain sections in rats treated with HANPs (G5) revealed some histopathological alterations including presence of large number of damaged neurons, moderate degenerating neurons, many apoptotic bodies, and moderate neuronal atrophy (Fig. 4E). As illustrated in Fig. 4F, the group treated with HANPs and CNPs together (G6) showed modest improvement in the brain sections, with a moderate number of injured neurons, moderately degenerating neurons, and a moderate neuronal atrophy and neurofibrillary degeneration. Treatment with HANPs and CUNPs (G7) showed mild degree of improvement with only moderate degenerating neurons, some apoptotic bodies and mild neuronal atrophy (Fig. 4G). The group treated with HANPs along with CNPs and CUNPs (G8), showed a more or less normal distribution of the neurons and nerve fibers with mild degenerating neurons and mild atrophy compared with control (Fig. 4H).

### Effect of CNPs, CUNPs and HANPs and their combination on the proliferating cell nuclear antigen immunoreactivity (PCNA-ir) in brain tissues

**Fig. 5:**
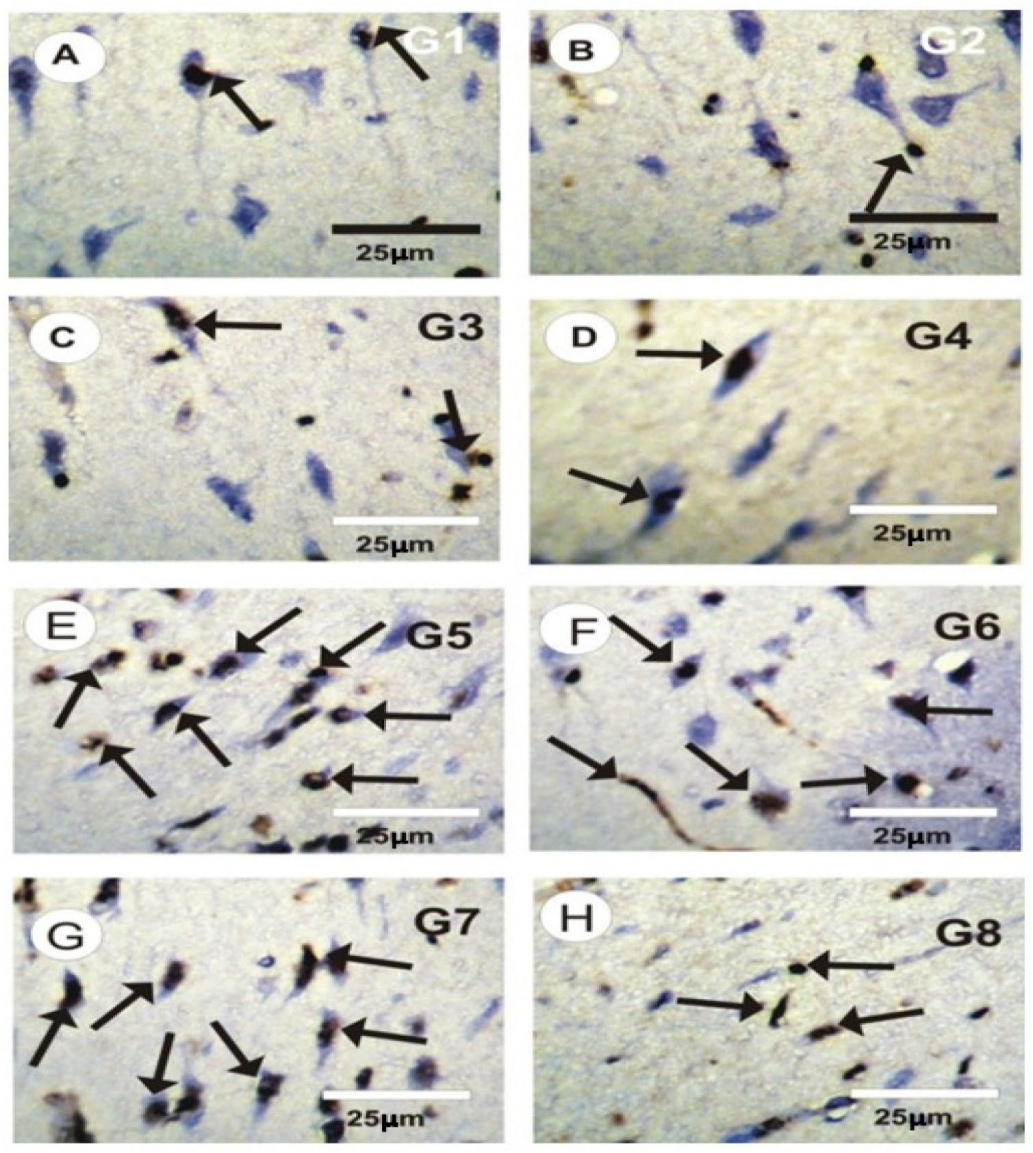
Photomicrographs of rat brain sections in the different experimental groups stained with PCNA-ir (arrows). (**A**–**D**) Mild positive reaction for PCNA-ir in neurons (microglia/macrophage) in brain cortex section in control (G1), in treated rats with CNPs (G2), with CUNPs (G3) and with CNPs and CUNPs (G4) groups. (**E**) Strong positive reactions (arrows) for PCNA-ir in the brain cortex sections in treated rat with HANPs. (**F**,**G**) Moderate positive reactions (arrows) for PCNA–ir in brain sections in treated rat with HANPs and CNPs or in treated rat with HANPs and CUNPs. (**H**) Mild positive reactions for PCNA–ir in brain sections in treated HANPs, CNPs and CUNPs (G8).

The brain tissues of control rats (G1), rats treated with CNPs (G2), CUNPs (G3) or both CNPs and CUNPs (G4) showed mild positive reaction for PCNA-ir in neurons (Fig. 5A–D). In contrast, strong PCNA immunoreactivity was found in the brain tissue of rats given HANPS (Fig. 5E). In comparison to control group, the intensity of PCNA-ir in brain tissues of HANPs treated group (G5) was significantly increased. In HANPs + CUNPs and HANPs + CNPs treated groups, moderate positive reactions for PCNA-ir were seen in the brain tissues (Fig. 5F,G). The brain sections of rats supplemented with a combination of HANPS, CNPS and CUNPs (G8) exhibited mild positive reactions for PCNA-ir in neurons (Fig. 5H).

## Discussion

The neurotoxic effects of HANPs and the possible mechanisms behind these effects are still unknown. A better understanding of these mechanisms is crucial to find the best methods of protection against HANPs neurotoxicity. In the present study, the neurotoxic potential of HANPs on the cerebral cortex was investigated at histological, biochemical, and molecular levels.

The present findings revealed that HANPs caused marked disruption in the cortical neurotransmitters, including decreased levels of Ach, DA, SER, EPI, and NOR. Multiple studies have confirmed the nanoparticle-induced deficiency in levels of neurotransmitters that leads to various disorders^16^. Dziendzikowska et al.^17^ reported that silver nanoparticles (AgNPs) alter neurotransmitters levels in hippocampus leading to synaptic degeneration and impairment of cognitive abilities.

Mitochondrial dysfunction, oxidative stress and inflammation are common pathways that are associated with neurotoxicity as well as neuronal dysfunction and the onset of neurodegenerative diseases^18^. Nanoparticles can penetrate the blood brain barrier and cause neurotoxicity through different mechanisms including increased oxidative stress resulting in mitochondrial dysfunction, neuroinflammation, apoptosis and neuronal death^19,20^.

In the present study, HANPs significantly inhibited expression of genes involved in mitochondrial biogenesis control namely; PGC-1α and mTFA. This indicates reduced mitochondrial biogenesis as well as mtDNA replication and transcription that could result in mitochondrial dysfunction and neuronal injury. Regarding CS activity, a biomarker for mitochondrial activity, the HANPS group also showed lower CS activity, indicating dysfunctional mitochondria and collapsed bioenergetics.

The underlying mechanism by which HANPs suppress the genes expression of brain mtTFA and PGC-1α as well as CS activity is unclear. We can assume that HANPs have the ability to penetrate the BBB and neuronal cells, leading to increased oxidative stress. Indeed, decreased mitochondrial biogenesis and activity in our study was accompanied with elevated levels of lipid peroxidation and oxidative DNA damage after HANPs exposure. HANPs led to significant inhibition of antioxidant enzymes; superoxide dismutase (SOD), glutathione peroxidase (GPx), catalase and decline of glutathione (GSH) total antioxidant capacity (TAC) levels. This deficiency in antioxidant defence system might be related to nitric oxide end products. The combination of reduced antioxidants and increased ROS aggravates oxidative stress caused by HANPs supplementation.

The effect of HANPs on mitochondrial function and oxidative status was investigated in previous studies. A recent study by Xia et al.^21^ revealed that HANPs increased mitochondrial-based pyroptosis and redox imbalance in the vascular smooth muscle cells. Baratli et al.^22^ demonstrated deleterious effects of NPs on mitochondrial respiratory chain complexes of middle aged rats. Also, it has been reported that HANPs lead to dose-dependent oxidative damage, genotoxicity and cytotoxicity in the human blood cells and a significant increase in 8-OH-dG levels. The rise in the frequencies of sister chromatid exchange, chromosome aberration rates and DNA damage was observed^23^. After the exposure to HANPs, Chen et al.^24^ also found that H_2_O_2_ and MDA levels were elevated and SOD and GSH levels were reduced.

Regarding the inflammatory status, HANPs exposed rats caused a marked increase in the levels of tumor necrosis factor-α (TNF-α) and interleukin-6 (IL-6). In line with these results, Velard et al.^25^ found that neutrophils treated with HANPs secrete cytokines (IL-1α and IL-8) and chemokines that lead to leukocyte chemotaxis. TNF-α has been linked to the enhancement of neutrophil recruitment into inflammatory sites. Also, it was documented that HANPs have immune-stimulatory potential in vitro and in vivo^26^.

P53 is a powerful apoptotic inducer under oxidative stress that is dramatically increased by HANPs. DNA damage and hypoxia can cause increased p53 protein expression. It has been demonstrated that HANPs increased intracellular ROS generation and stimulated p53, which may cause DNA damage in human breast cancer cells^27^. In our study, P53 may play a critical role in apoptosis after HANPs treatment by down-regulating the anti-apoptotic Bcl-2 family expression^28^.

The neurotoxic effects of HANPs were confirmed by histopathological investigation of brain tissue. HANPs exposure provoked several alterations in the structure of neurons and nerve fibers in the form of degenerated neurons, neuronal atrophy, a lot of damaged neurons and apoptotic bodies. Also, findings showed high intensity of PCNA, indicating increased proliferation associated with neuroinflammation due to HANPs neurotoxicity.

Collectively, HANPs have possible neurotoxic effects and can significantly influence brain antioxidant systems, mitochondrial biogenesis, neuronal structure and elicit neuro-inflammation and apoptosis. Given the widespread use of HANPs in medicine, the need for protective approaches against their toxicity is of critical importance. In this research, we investigate the protective effects of chitosan nanoparticles (CNPs) or nanocurcumin (CUNPs) alone or in combination when co-supplemented with HANPs.

The results indicated the potential protective effects of both CNPs and CUNPs against HANPs-induced neurotoxicity with noticeable therapeutic benefits by using CNPs either alone or in combination with CUNPs. Chitosan and curcumin are well known naturally occurring compounds that are widely used for human dietary use with powerful anti-oxidants and anti-inflammatory effects. The nanoparticles of these two compounds also have more potent effects with increased bioavailability^29,30^.

The protective effects of CNPs and CUNPs are associated with significant correction of brain neurotransmitters; Ach, DA, SER, EPI and NOR. However, their levels still lower than control values. Increased levels of neurotransmitters observed might be due to inhibition of monoamine oxidase enzyme (MAO) activity^31^. Similarly, Fan et al.^32^ showed that curcumin was an effective antidepressant and prevented excessive synaptic loss in a rat model of depression. Salah et al.^33^ demonstrated that nanocurcumin significantly improved the levels of neurotransmitters; ACh, serotonin, and dopamine as well as AchE protein content in the brain tissues. After severe spinal cord injury, it has been found that CNPs successfully restored nerve impulse transmission and they return the improvement to chitosan’s membrane sealing properties^34^.

At the molecular level, the current research showed that HANPs combined with CNPs and/or CUNPs improve the brain expression of PGC-1α and mTFA genes, indicating restoration of mitochondrial biogenesis and function with the best effect found in rats co-supplemented with CNPs alone or combined with CUNPs. CS activity was also increased in CUNPs alone group or in combination with CNPs when co-supplemented with HANPs. In addition, our findings clearly demonstrated that CNPs and CUNPs improved the antioxidant status of brain tissues by enhancing antioxidant enzymes, elevating TAC and glutathione replenishment. They inhibit NO production, as evidenced by lower nitrite levels. These beneficial effects on antioxidant and free radical levels led to the correction of oxidative stress status in brain tissues, as indicated by reduced levels of MDA and 8-OH-dG. Regarding cytokines levels, treatment with CNPs or CUNPs significantly reduced the increased levels of P53, TNF-α and IL-6 in the brain cortex that were induced by HANPs. When CNPs or CUNPs were combined, the results showed strong synergistic effects.

In accordance with these results, it has been found that curcumin could modulate the functions of mitochondria by altering mitochondrial ROS production and transcription factors activity, regulating mitochondrial proteins expression^35^. Ahmed-Farid et al.^36^ reported that CUNPs caused pronounced decrease in 8-OHdG level as well as reduced oxidative and nitrosative stresses. It has been found that nano-curcumin effectively managed experimental type 1 diabetes mellitus by preventing streptozotocin-induced inflammation and apoptosis and reducing the level of 8-OHdG in pancreatic beta cells^37^. Moreover, Ragusa et al.^38^ demonstrated that CNPs potentially exerted neuroprotective effects in dopaminergic SH-SY5Y cells against oxidative stress. A recent study by Mosa et al.^39^ also revealed that coadministration of chitosan nanoparticles and/or curcumin nanoparticles successfully ameliorate the toxic effects of HANPs by improving antioxidant parameters and suppressing levels of P53, TNF-α, and interleukin-6 in the heart.

The protective impact of CNPs and CUNPs was confirmed by histopathological analysis. In our study, CUNPs co-supplementation showed mild degree of improvement with only moderate degenerating neurons, while CNPs co-supplementation alone or in combination with CUNPs showed a more normal distribution of neurons and nerve fibers with only mild degenerating neurons and mild atrophy. In addition, the immunoreactivity to PCNA was significantly decreased in the brain tissues of rats received CNPs or CUNPs with HANPs. The co-supplementations with both protective agents have better effect.

In conclusion, long-term exposure to oral HANPs has neurotoxic effects at various levels involving histological structure, immunohistochemical reactivity towards PCNA, neurotransmitters, redox status, DNA damage, cytokine production, and mitochondrial biogenesis. The study provides evidence of the beneficial effects of CNPs and CUNPs alone or in combination against the neurotoxicity caused by HANPs. Our findings require further proof of evidence by conducting future investigations to study the direct effects of HANPs and or CNPs and CUNPs on the mitochondrial preparations and conducting analysis of mitochondrial membrane potential and oxygen consumption rate which will provide the functional evidence of alterations of mitochondrial functions.

## Materials and methods

### Materials

To prepare hydroxyapatite nanoparticles (HANPs), sodium carbonate Na_2_CO_3_ and sodium hydroxide (NaOH) reagents were purchased from Riedel-de-Haën, Germany and El-Nasr pharmaceutical chemicals Co., Egypt, respectively. The chitosan nanoparticles (CNPs) and curcumin nanoparticles (CUNPs) were obtained from Cognis Co., Norway and Nanotech Egypt for Photo Electronics, respectively. Analytical grade materials were utilized throughout the experiment.

### Synthesis and characterization of HANPs using TEM

The HANPs were synthesized from natural HP extracted from bovine bone. The detailed methods of preparation and characterization of the HANPs were explained in our previous papers^9,10^. The organic matter was extracted from bovine bone mechanically by removing the salt tissue adhered to the bone and treatment with 30% sodium carbonate (Na_2_Co_3_). Then, calcinations of alkali-treated bone were done and an electrically heated furnace with two holes was used for this technique. The temperature of furnace was progressively raised at a rate of 5 °C/min in a strong air steam. A platinum disc was employed to insert the bone regments (cutoff). After raising the temperature to 800 °C, it was soaked for 2 h to obtain the ultimate firing temperature. Grinding of the treated cut off bones was accomplished using grinding apparatus (Gilson company Inc. model USA, No Lc-91). Nanohydroxyapatite was obtained from crushed bone by heating with 2M NaOH. The finalized HAPNPs were placed into previously sterilized glass containers and subjected to 2.5 M rad of gamma radiation to achieve sterilization.

By using a high resolution transmission electron microscope (JEOL JEM 2100 Plus), all NPs were characterized. The NPs were put on carbon-coated copper grids, dried for 5 min at room temperature and 2.0% (w/v) phosphor-tungstic acid was added for observation. The HR-TEM can perform multi-position acquisitions automatically. The acceleration voltage can be set between 80 and 200 kV based on the specimen’s type and thickness [^40,41^.

### Animals and experimental design

Eighty adult male Wistar rats weighing 170–175 g were obtained from the animal house of Faculty of Medicine, Alexandria University, Egypt. Rats were maintained in a controlled environment with room temperature of 25 °C and natural light–dark cycle (12/12 h) and received diet and water ad libitum. The current study adheres to the ARRIVE Guidelines for reporting in vivo experiments^42^. All procedures and experimental protocols were approved by and carried out based on the Alexandria University Institutional Animal Care and Use Committee (AU-IACUC) guidelines (Approval number: AU0122212232). Throughout the experimental period, every effort was made to minimize the distress of the rats.

Rats were divided into 8 groups, 10 rats each. Group 1 (Control): Normal control rats, Group 2 (CNPs): normal rats received 280 mg/kg of CNPs,^43–45^, Group 3 (CUNPs): normal rats received 15 mg/kg of CUNPs^46^, Group 4 (CNPs + CUNPs): normal rats received mixture of CNPs (280 mg/kg) and CUNPs (15 mg/kg), Group 5 (HANPs): rats were received 300 mg/kg of HANPs^47^, Group 6 (HANPs + CNPs): rats were received 300 mg/kg of HANPs and co-supplemented with 280 mg/kg of CNPs, Group 7 (HANPs + CUNPs): rats were received 300 mg/kg of HANPs and co-supplemented with 15 mg/kg of CUNPs, and Group 8 (HANPs + CNPs + CUNPs): rats were received 300 mg/kg of HANPs and co-supplemented with mixture of 280 mg/kg of CNPs and 15 mg/kg CUNPs. For 45 days, the animals were given their respective doses daily via oral gavage.

### Samples collection and tissue preparations

At the conclusion of the experiment, rats were sacrificed under anesthesia (isoflurane inhalation). The brain of all groups was removed and cleaned with (0.9%) saline solution. After separation of two hemispheres, one was used to obtain the cerebral cortex, while the other was fixed in normal formalin for histological investigation. The cerebral cortex was divided into three sections. The first section was used for RNA extraction to study the gene expression, the second was used for DNA isolation to measure 8-hydroxy deoxyguanosine (8-OHdG), and the third was homogenized in phosphate buffered saline (1:9). After centrifugation at 10,000×*g* for 20 min at 4 °C, the supernatants were stored at – 80 °C.

### Assays of neurotransmitters

The tissue levels of acetylcholine (Ach), dopamine (DA), serotonin (SER), epinephrine (EP) and norepinephrine (NE) were determined using ELISA kits (Cloud-Clone Corp. Houston, USA).

## Assay of malondialdehyde (MDA) and nitric oxide end products (*NOx*)

The brain levels of the lipid peroxidation marker, MDA were measured according to the method of Tappel and Zalkin^48^, whereas the levels of nitric oxide end products; nitrite and nitrate (NOx) were determined by Montgomery and Dymock’s method^49^. MDA was quantified by reaction with 2-thiobarbituric acid (4,6-dihydroxypyrimidine-2-thiol; TBA) to produce a pink colored complex, which was measured at 532 nm.

### Assay of 8-hydroxy deoxyguanosine (8-OHdG)

The genomic DNA was isolated using GeneJet Genomic DNA purification kit (Thermo Scientific, USA). In brief, a glass Teflon homogenizer was used to break up 20 mg of brain tissue into small pieces. 180 μL of Digestion Solution and 20 μL of Proteinase K Solution were added and the samples were kept in an incubator at 56°C. After adding 20 μL of RNaseA Solution, 200 μL of Lysis Solution and 400 μL of 50% ethanol, the lysate was placed inside a Genomic DNA Purification Column and centrifuged at 6000×*g* for 1 min was done. To elute genomic DNA, 200 μL of elution buffer was added and the extracted DNA was kept in storage at – 20 °C. For enzymatic digestion, the isolated DNA was diluted in 200 μl of 10 mM Tris/HCl, 0.1 mM EDTA, and 100 mM NaCl (pH 7.0) after being twice cleaned with 70% ethanol and dried. A total of 200 μg of DNA was treated for 1 h at 37 °C with 100 units of DNase I in 40 μl Tris/HCl 10 mM and 10 μl of 0.5 M MgCl_2_ (final concentration of 20 mM). After adding 15 μl of sodium acetate 0.5 M (pH 5.1) to lower the pH of the reaction mixture, 10 μl (5 units) of nuclease P1 and 30 μL of 10 mM ZnSO4 were added. Following the addition of 20 μl of alkaline phosphatase and pH adjustment with 100 μl of 0.4 M Tris/HCl (pH 7.8), the samples were incubated for 30 min at 37 °C, boiled for 10 min. DNA hydrolysate was used to measure 8-OHdG by 8-OHdG ELISA kit (Abcam, Cambridge, UK).

### Assay of antioxidants

After precipitation by meta-phosphoric acid, reduced glutathione (GSH) content was assayed^50^. The 5,5′-dithiobis-(2-nitrobenzoic acid) (DTNB) oxidation of GSH to produce GSSG and 5-thio-2-nitrobenzoic acid (TNB) served as the basis for the assay. At 412 nm, the rate of TNB production was measured, and it was proportionate to the amount of GSH in the sample. The activity of glutathione peroxidase (GPx) in cerebral cortex was determined by Chiu et al.^51^ method. Superoxide dismutase (SOD) activity and glutathione S-transferase (GST) were measured using methods of Mishra and Fridovich^52^ and Habig et al.^53^, respectively. The assay method of SOD activity includes the marked suppression of epinephrine auto-oxidation to adrenochrome in an alkaline media (pH 10.2) by SOD. Tissue supernatant was mixed with epinephrine, and the extinction coefficient change was measured at 480 nm. GST activity analysis based on catalysis of the conjugation of glutathione in the first step of mercapturic acid synthesis. By a UV-Double Beam Spectrophotometer, the absorbance was detected at 310 nm. Catalase (CAT) activity was assayed in cerebral cortex depending on hydrogen peroxide (H_2_O_2_) decomposition Luck method^54^. The rate at which H_2_O_2_ degraded was used to assess CAT activity spectrophotometrically at 240 nm. The levels of total antioxidant capacity (TAC) were assayed based on the method of Koracevic et al.^55^. The above assays were determined according to the manual instruction of Biodiagnostic Kit, Egypt.

### Assay of tumor necrosis factor-alpha (TNF-α), Interleukin-6 (IL-6) and tumor suppressor gene p53

The tissue contents of TNF-α, IL-6 and P53 were quantified using specific ELISA kits purchased from Abcam Co., UK, Kamiya Biomedical Co., USA, and Active Motif Co., USA, respectively based on the manufacturer’s instructions.

### Citrate synthase (CS) activity

The oxidative capacity and activity of mitochondria were evaluated by determination of citrate synthase enzyme activity. Through the formation of –SH group by using the reactive Ellman reagent (DTNB) and measuring the change of absorbance value at 412 nm, the activity of citrate synthase (CS) was quantified as Unit/mg protein^56^.

### qRT-PCR analysis of mRNA expression of peroxisome proliferator activator receptor gamma coactivator 1α (PGC-1α) and mitochondrial transcription factor A (mTFA)

Total RNA was extracted from cerebral cortex by RNeasy Mini Kit (Qiagen, Germany). 30 mg of sample was used, mixed and homogenised after addition of 600 μl Lysis buffer, then centrifuged at maximum speed for 3 min. The clear supernatant was combined with one volume of 70% ethanol and a volume of up to 700 µl was moved to an RNeasy spin column, which was then put in a 2 ml collecting tube and centrifuged for 15 s at 12,000 rpm. 50 μl RNase-free water was added to the spin column membrane, and it was centrifuged for 1 min at 12,000 rpm to elute the RNA. The RNA sample was kept at -20°C and Nano drop verified the integrity of RNA. The reverse transcription into cDNA was carried out using QuantiTect Reverse Transcription Kit following the manufacturer instructions. Using specific primers, quantitative PCR was used to determine the relative expression of PGC-1 and mTFA. The Rotor-Gene SYBR Green PCR Kit (Qiagen®, Germany) was used to perform PCR. The PCR program starts with 10 min of denaturation at 95 °C, followed by 45 rounds of 3 steps, including 15 s of denaturation at 95 °C, 15 s of annealing at 55 °C and 15 s of extension at 60 °C. The relative expressions of PGC-1α and mTFA were assessed relative to the expression of GAPDH as a reference gene, ΔΔCt method or Livak method was used to calculate PGC-1α and mTFA gene expressions^57^.

**Table:** 

Gene	Primer sequence	
PGC-1α	F:	5′-GTGCAGCCAAGACTCTGTATGG-3′
	R:	5′-GTCCAGGTCATTCACATCAAGTTC-3′
mTFA	F:	5′-CCCTGGAAGCTTTCAGATACG-3′
	R:	5′-AATTGCAGCCATGTGGAGG-3′
GAPDH	F:	5′-GGGTGTGAACCACGAGAAATA-3′
	R:	5′-AGTTGTCATGGATGACCTTGG3′

### Histopathological analysis

Following standard procedures, the fixed brain was dehydrated in a graded series of ethanol and embedded in paraffin. Some paraffin sections were stained with haematoxylin and eosin according to the method of Nobakht et al.^58^, while the remainder was used for PCNA immunohistochemical staining as described by Tousson et al.^59^.

### PCNA immunoreactivity (PCNA-ir)

Avidin–Biotin-Peroxidase (ABC) immunohistochemical method (Elite-ABC, Vector Laboratories, CA, USA) was used to investigate the distribution of PCNA subunits in the brain sections. After deparaffinization, sections were rehydrated and rinsed in PBS three times for five seconds. For blocking of peroxidase activity, sections were incubated in 0.3 percent H_2_O_2_ in methanol. Sections were then incubated with anti-PCNA monoclonal antibody. After washing with PBS, sections were incubated with biotinylated secondary antibody, followed by streptavidin peroxidase and the antibody-peroxidase complex was detected using diaminobenzidine (DAB chromogen). After staining with haematoxylin, sections were dehydrated in a series of graded alcohols, rinsed in xylene, and cover-slipped.

### Statistical analysis

The data were analyzed statistically using the general linear model (GLM) produced by Statistical Analysis Systems Institute (SAS, 1998). Data were expressed as means ± SD. To determine whether the discrepancies between the means were significant, Tukey Post Hoc Test was applied. Values of p < 0.05 were considered statistically significant.

## Data Availability

The datasets generated during and/or analysed during the current study are available from the corresponding author on reasonable request.
